# The Ottawa Charter: Indigenous sovereignty, resistance, and health promotion at 40

**DOI:** 10.1093/heapro/daaf195

**Published:** 2025-11-24

**Authors:** Raglan Maddox, Chase Kornacki, Shane Kawenata Bradbrook, Tom Calma

**Affiliations:** Yardhura Walani, National Centre for Epidemiology and Population Health, Bagumani (Modewa) Clan, The Australian National University, Canberra, ACT 2600, Australia; Biology and Environment, Australian National University (ANU) College of Medicine, 54 Mills Rd, Canberra, ACT 2602, Australia; Navajo Nation, Sheepsprings Chapter, Shiprock Agency, Sheepsprings, NM 87323, United States; Southern California American Indian Health Center, 4058 Willows Road, Alpine, CA 91901, United States; Ngāi Tāmanuhiri, Rongowhakaata, Ngāti Kahungunu, Aotearoa (New Zealand), PO Box 7343, Wellington 6242, New Zealand; Elder from the Kungarakan Tribal Group and a Member of the Iwaidja, PO Box 4273, Canberra, ACT 2601, Australia; University of Sydney, Camperdown, NSW 2050, Australia

Contribution to Health Promotion

- The Ottawa Charter is a powerful guide for fair and healthy societies, but inequities and industry-generated harms persist.
- Tobacco and nicotine industries continue to exploit colonial systems, targeting Indigenous peoples and undermining sovereignty and the right to health.
- Indigenous communities demonstrate strong leadership, including youth leadership, through truth-telling, art, and community action that resist commercial industry control, disease and death.
- Health promotion must centre sovereignty, justice, and equity to confront racism, sexism, ageism, ableism, colonialism, and digital exploitation.
- The future of health promotion lies in abolitionist and structural reform, building fair and healthy futures for all, free from industry-generated harms.

## INTRODUCTION: CELEBRATING AND CHALLENGING THE OTTAWA CHARTER FOR HEALTH PROMOTION

Forty years after the Ottawa Charter for Health Promotion was adopted, its influence endures as a powerful framework for advancing equity, justice, health and wellbeing (World Health Organization 1986, 2016). The Charter’s vision of enabling people to take control of their health, through building healthy public policy, creating supportive environments, strengthening community action, developing personal skills, and reorienting health services, has shaped global, national, regional and local practice, and remains foundational today (World Health Organization 1986, 2016). It has inspired generations of researchers, practitioners, advocates and community members to take seriously the task of linking social justice and health.

Forty years on from the Ottawa Charter for Health Promotion, the call to ‘reorient health systems’ has continued to expand beyond hospitals and clinics to include digital, ecological, and economic systems (World Health Organization 1986, 2016, Maddox and Bradbrook 2025, Purnat et al. 2025, Thomas et al. 2025). The Charter’s five action areas (building healthy public policy, creating supportive environments, strengthening community action, developing personal skills, and reorienting health services) remain critical signposts for a world now shaped by commercial and algorithmic determinants of health (World Health Organization 1986).

Yet, the structural drivers of inequity persist, particularly for Indigenous peoples (Crocetti et al. 2022, Maddox and Bradbrook 2025, Purnat et al. 2025). Tobacco and nicotine industries continue to exploit colonial and racial structures, perpetuating addiction, disease, and death while undermining sovereignty, self-determination and the human right to health (Rose et al. 2024, Maddox and Bradbrook 2025). The Ottawa Charter was groundbreaking in its articulation of structural change, but it is now challenged by the scale and sophistication of commercial determinants of health (Thomas et al. 2024). To remain relevant, health promotion must celebrate its legacy while facing these new and evolving realities (World Health Organization 1986, 2016, Thomas et al. 2024, 2025, Purnat et al. 2025).

## STRUCTURAL HARMS AND THE COMMERCIAL DETERMINANTS OF HEALTH

As the commercial determinants of health and wellbeing become increasingly visible, the Ottawa Charter must continue to grow and evolve, centring sovereignty, youth leadership, and abolitionist responses to industry harms (Thomas et al. 2024, 2025, Purnat et al. 2025). Commercial actors, including the tobacco and nicotine industry, operate within markets and actively shape political, legal, and cultural systems to sustain profit, addiction, disease, and death (Thomas et al. 2024, Maddox and Bradbrook 2025). For Indigenous peoples, these strategies extend and intensify colonial legacies of dispossession, control, and erasure. These harms reflect racial capitalism and state-enabled corporate influence, entrenching inequity across generations (Crocetti et al. 2022, Odo et al. 2025).

For example, the global expansion of e-cigarettes, heated tobacco and other non-therapeutic nicotine products demonstrates how industries frame themselves as ‘harm reduction’ partners while continuing to addict new generations. These narratives mirror earlier tactics used to suppress Indigenous knowledge systems, silence critical scholarship, and normalize dependence. Without centring sovereignty and justice, health promotion risks being co-opted by the very industries it seeks to resist.

As highlighted by Thomas et al. (2025), health promotion must now confront the commercial and digital determinants of health. Algorithms, monetization models, and data systems have become new vectors of inequity, shaping what information, products, and ideologies people encounter (Crocetti et al. 2022, Purnat et al. 2025, Thomas et al. 2025). The same systems of profit and surveillance that amplify commercial tobacco and alcohol marketing are those that now curate the very health information people trust. Naming and confronting these digital architectures is essential to fulfilling the Ottawa Charter’s unfinished business of enabling and mediating for equity (Purnat et al. 2025, Thomas et al. 2025).

## FROM PARTICIPATION TO LEADERSHIP: INDIGENOUS-LED HEALTH PROMOTION

Drawing on Indigenous-led experiences in Australia, Aotearoa New Zealand, Turtle Island, and beyond, we illustrate how shifting from participation to leadership can advance truth-telling, accountability, and futures free from industry exploitation and control (Maddox and Bradbrook 2025, Maddox and Morton Ninomiya 2025). Health promotion must move beyond consultation or symbolic inclusion of Indigenous peoples. True sovereignty requires decision-making power, resource control, and the authority to set the terms of engagement (Whyte et al. 2018, Colonna et al. 2020, Maddox and Morton Ninomiya 2025).

Youth are not simply the ‘future’ but are already present-day leaders, carrying languages, cultures, and responsibilities forward, mobilizing creativity, ancestral continuity, and collective strength to resist industry harms and promote health and wellbeing (United Nations 1989, 2007, Maddox and Bradbrook 2025). Importantly, leadership is also expressed in the everyday, by being Indigenous young peoples, having fun, and doing deadly*(In Aboriginal and Torres Strait Islander cultures, deadly is a positive term used to describe something excellent, awesome, great, strong, or impressive.) things, youth embody cultural strength and possibility. Sovereignty is lived. Everyday acts of joy, cultural continuity, and refusal of deficit framings are active forms of leadership and resistance (Corntassel and Scow 2017, Heid et al. 2022). As Ratima et al. (2019) emphasize, self-determination and sovereignty must anchor healthy public policy. Indigenous governance is not simply a matter of inclusion but of (re)centring authority and knowledge systems within decision-making. (Re)engaging the Adelaide Recommendations (1988) reminds us that the unfinished work of reconciliation and Treaty in Australia is inseparable from the health promotion project itself (World Health Organization 1988). The TEPOC Youth Advisory Council (YAC) in California provides a compelling example. Tribal youth are driving programme design, implementation, and evaluation. Their leadership demonstrates how Indigenous governance can transform prevention strategies, embedding cultural continuity and advocacy into the fabric of health promotion (Frazier et al. 2025).

## CELEBRATING INDIGENOUS EXEMPLARS

Celebration is as vital as critique. The Ottawa Charter at 40 is a moment to recognize how Indigenous leadership has advanced health promotion through innovation, resilience, and creativity.

**Figure 1.**
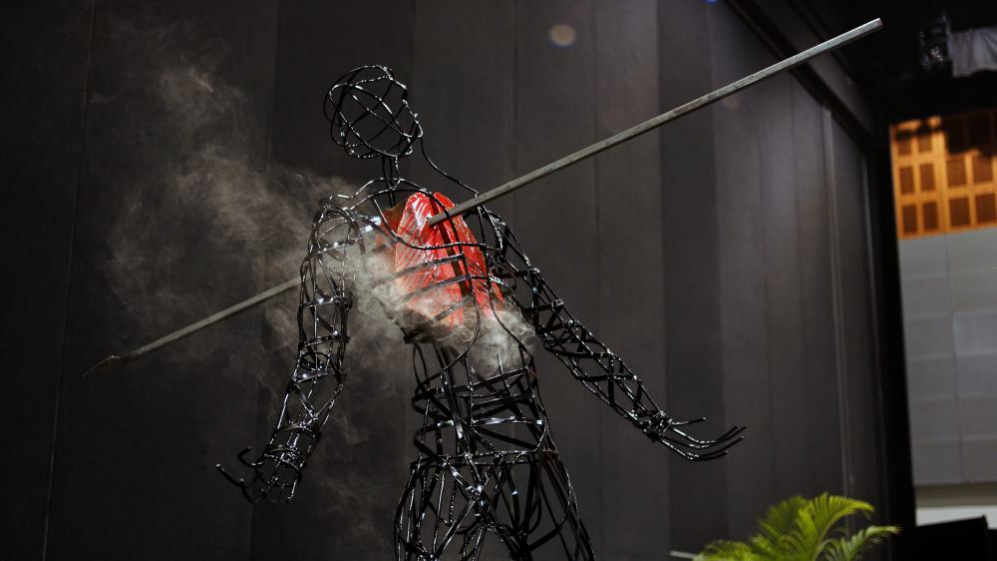
Kilum Murubul Jum (Death by Smoke) by Left ear experiment—a sculpture embodying indigenous truth-telling, remembrance, and resistance to industry harm.

- In Aotearoa New Zealand, the Māori Killer and Extinct Tobacco Industry campaigns disrupted public narratives and (re)framed commercial tobacco as a colonial agent of harm. These creative, bold campaigns shifted discourse from individual behaviour to structural accountability and justice.
- The Funeral for the Tobacco Industry, also in Aotearoa New Zealand, stands as a symbolic act of collective resistance and truth-telling, demonstrating how ritual and community action can redefine what health promotion looks like.
- In Australia, Kilum Murubul Jum (‘Death by Smoke’) by the Left Ear Experiment (*Fig. 1*) is a SWELL Indigenous Artist Award winning sculpture that embodies truth-telling, grief, and resilience in the face of ongoing tobacco and nicotine industry violence. As an arts-based intervention, it expands health promotion beyond policy and clinics into culturally specific spaces, places, and times. This recognizes that health, identity, and meaning are always situated within temporal, spatial, and cultural contexts (i.e. time, place and space specific).
- The Tackling Indigenous Smoking programme in Australia has supported significant declines in prevalence among Aboriginal and Torres Strait Islander peoples. Despite ongoing challenges and an ever-active tobacco and nicotine industry, it is a reminder of what is possible when Indigenous-designed, community-led programmes are resourced and sustained (Colonna et al. 2020).
- On Turtle Island, TEPOC’s YAC created a model of intergenerational leadership in commercial tobacco and nicotine prevention. By designing and advocating their own strategies, Tribal youth demonstrate that community action, when paired with sovereignty, can disrupt industry control (Frazier et al. 2025).

Together, these exemplars demonstrate that Indigenous peoples are active architects of futures free from industry exploitation. They also highlight the diverse modalities through which sovereignty, resistance, and resilience are expressed, including art, languages, cultural continuity, programmatic achievement, and governance (Colonna et al. 2020, Heid et al. 2022, Frazier et al. 2025).

## POLICY INCOHERENCE AND SUPPLY REDUCTION

At the same time, policy incoherence persists. Maintaining widespread retail availability of tobacco and nicotine products while simultaneously advancing strong tobacco resistance measures creates contradictions that weaken enforcement, sustain oversupply, and leave communities vulnerable to ongoing industry harms and interference (Dessaix et al. 2025). Australia’s experience is instructive: despite comprehensive tobacco resistance policies, the unchecked proliferation of retail outlets has normalized tobacco access, undermined equity, and compromised the human right to health (Dessaix et al. 2025).

Addressing these contradictions requires closing regulatory gaps but actively enforcing policies and reducing product availability (Dessaix et al. 2025, Maddox and Bradbrook 2025). This requires nationally consistent licencing schemes, capping, and reducing outlets, phasing down sales as well as track and trace systems to monitor and evaluate health promotion activities ensuring measures align with the World Health Organization Framework Convention on Tobacco Control (WHO FCTC) commitments and uphold the human right to health (World Health Organization 2003, Dessaix et al. 2025, Maddox and Bradbrook 2025). In Aotearoa New Zealand, the recent repeal of the world-leading Smokefree 2025 measures, including denicotinization, retail reduction, smoke-free generation policy, and Māori governance demonstrates the fragility of progress and the urgency of sustained abolitionist strategies. Health promotion cannot assume progress is linear; ongoing vigilance, resistance, and community-led programmes, policies and advocacy remain essential.

## TOWARDS HEALTH JUSTICE: EXPANDING THE CHARTER

To realize the next evolution of the Ottawa Charter, we argue that health promotion must embed Indigenous epistemologies, Indigenous Data Sovereignty, and intergenerational leadership. Drawing on Dr. Rhys Jones' advice, it must foreground intersectionality and commit to being explicitly anti-racist, anti-sexist, anti-homophobic, anti-ageist, and anti-ableist. Health promotion cannot be neutral in the face of oppression. Neutrality only reinforces existing inequities. A key lesson from 40 years of the Ottawa Charter is that health promotion must be politically courageous, ‘naming and taming’ the forces that shape public health agendas, whether they be the tobacco industry or platform economies. Reaffirming the Charter’s ethos of social justice means exposing the corporate appropriation of health promotion language, the transformation of public values into market slogans. Health justice is inseparable from racial justice, demanding that we disrupt the systems that sustain oppression and inequity (Maddox and Bradbrook 2025). For Indigenous peoples, sovereignty is lived daily in acts of cultural continuity, joy, and refusal of deficit framings, reminding us that thriving itself is a form of resistance (Ratima et al. 2019, Maddox and Bradbrook 2025, Maddox and Morton Ninomiya 2025). Confronting intersecting systems of oppression is not peripheral but central to dismantling the conditions that allow commercial industries to profit and thrive by harming Indigenous peoples (Crocetti et al. 2022, Thomas et al. 2024, Maddox and Bradbrook 2025).

Health promotion can no longer treat the information environment as neutral. The digital sphere now determines whose truths are amplified and whose are buried (Purnat et al. 2025). The Charter’s call to create supportive environments extends to digital architectures, requiring policies that prioritize health, trust, and truth over profit (World Health Organization 1986, 1988, Purnat et al. 2025, Thomas et al. 2025).

This is what we mean by moving from health promotion to actively include health justice: a field that is unapologetically political, that resists co-option, and that builds alliances across movements for Indigenous rights, gender equity, racial justice, and environmental survival (Whyte et al. 2018, Ratima et al. 2019, Maddox and Bradbrook 2025, Maddox and Morton Ninomiya 2025).

## HEALTH PROMOTION HOOKS: FROM OTTAWA TO THE FUTURE AND BEYOND

To align with both the legacy and future of the Ottawa Charter, we call for action across its five domains:

- Build healthy public policy: Embed Indigenous governance, self-determination such as Indigenous Data Sovereignty Principles into law and policy; adopt national tobacco licencing, cap and reduce retail outlets, and resource youth governance and advisory groups, such as TEPOC, to inform and monitor these changes (Colonna et al. 2020, Frazier et al. 2025).
- Create supportive environments: Learn from community-driven programmes such as Tackling Indigenous Smoking and TEPOC’s youth-led education campaigns, which build resilience and cultural pride while actively shifting norms (Colonna et al. 2020, Frazier et al. 2025).
- Strengthen community action: Resource Indigenous- and youth-led initiatives, from art and health science communications [Kilum Murubul Jum (‘Death by Smoke’), Māori campaigns, the Funeral for the Tobacco Industry] to structured governance (TEPOC YAC) that challenge industry harms while sustaining languages, cultures, health and wellbeing (Colonna et al. 2020, Frazier et al. 2025, Odo et al. 2025).
- Develop personal skills: Prioritize Indigenous data sovereignty, youth training and development, and Indigenous evaluation frameworks that build leadership, and resistance capacities (Ratima et al. 2019, Heid et al. 2022, Frazier et al. 2025, Maddox and Bradbrook 2025, Maddox and Morton Ninomiya 2025).
- Reorient health services: Position health promotion as explicitly anti-oppressive and justice-based, ensuring health systems align with Indigenous epistemologies and intergenerational equity (Ratima et al. 2019).

## CONCLUSION: OTTAWA AT 40 AND BEYOND

A future free from industry control and industry generated harms must also mean a future free from algorithmic manipulation and digital disinformation (Purnat et al. 2025). The same principles that guided tobacco control: transparency, regulation, and accountability must now be applied to digital and commercial platforms (World Health Organization 1986, 2003, United Nations 2007). Reaffirming the Ottawa Charter at 40 means resisting the old and new colonialisms of industry and data, and grounding health promotion in Indigenous sovereignty, ecological justice, and collective agency (Whyte et al. 2018, Maddox and Morton Ninomiya 2025, Thomas et al. 2025).

The Ottawa Charter at 40 provides an opportunity to celebrate its legacy but to acknowledge its unfinished business (Thomas et al. 2025). The next phase requires health promotion to mature into health justice: demanding accountability from states and corporations, centring sovereignty, and resourcing youth leadership to improve health and wellbeing (Heid et al. 2022, Thomas et al. 2024, Maddox and Bradbrook 2025).

We also recognize that Health Promotion International demonstrates active leadership in this space by refusing to publish research funded by the tobacco, alcohol, ultra-processed food, gambling, arms/weapons, or fossil fuel industries, or by organizations financed by these sectors (Health Promotion International 2025). This editorial standard reflects a commitment to health justice and to protecting the integrity of public health and health promotion scholarship from commercial influence.

If the Charter’s first four decades established the foundations of health promotion, the next four must focus on abolitionist strategies to dismantle industry generated harms, power and structural oppression (Maddox and Bradbrook 2025, Thomas et al. 2025). Futures free from industry control are already being built in communities of practice, including Health Promotion International, and across Australia, Aotearoa, Turtle Island, and beyond (Whyte et al. 2018, Ratima et al. 2019, Crocetti et al. 2022, Heid et al. 2022, Health Promotion International 2025). Health promotion must learn from, celebrate, and amplify these movements (World Health Organization 1986, 1988, Purnat et al. 2025, Thomas et al. 2025).

Only then can the Charter’s promise of equity and wellbeing be realized in ways that honour sovereignty, resist exploitation, and sustain generations to come. Health justice demands that we refuse deficit narratives and confront the systems of oppression themselves, unsettling the very conditions that enable industry exploitation and structural violence (Kickbusch et al. 2016, Thomas et al. 2024, Maddox and Bradbrook 2025).

## Data Availability

No data used in this Editorial.
